# When and how teachers intervene in group discussions on experiences from practice in postgraduate medical education: an interactional analysis

**DOI:** 10.1007/s10459-022-10122-w

**Published:** 2022-06-20

**Authors:** Marije van Braak, Mike Huiskes, Mario Veen

**Affiliations:** 1 Erasmus Medical Centre, Rotterdam, The Netherlands; 2 Rijksuniversiteit Groningen, Groningen, The Netherlands; 3 Utrecht University, Trans 10, 3512 JK Utrecht, The Netherlands

**Keywords:** Conversation analysis, First teacher intervention, Moderating actions, Expert actions, Evaluating actions, Group discussion, Postgraduate medical education

## Abstract

Medical educators constantly make decisions on when and how to intervene. Current literature provides general suggestions about types of teacher interventions. Our study aims to specify that knowledge by describing in detail the actions teachers do when intervening, the interactional consequences of those actions, and how these relate to teacher roles in group discussions. We collected all first teacher interventions (n = 142) in 41 videorecorded group discussions on experiences from practice at the Dutch postgraduate training for General Practice. We analyzed the interventions using Conversation Analysis. First, we described the timing, manner, actions, and interactional consequences of each intervention. Next, we inductively categorized actions into *types of actions*. Finally, we analyzed the distribution of these types of actions over the group discussion phases (telling, exploration, discussion, conclusion). First teacher interventions were done at observably critical moments. Actions done by these interventions could be categorized as moderating, expert, and evaluating actions. Moderating actions, commonly done during the telling and exploration phase, are least directive. Expert and evaluator actions, more common in the discussion phase, are normative and thus more directive. The placement and form of the actions done by teachers, as well as their accounts for doing those, may hint at a teacher orientation to intervene as late as possible. Since the interventions are occasioned by prior interaction and responded to in different ways by residents, they are a collaborative interactional accomplishment. Our detailed description of how, when and with what effect teachers intervene provides authentic material for teacher training.

## Introduction

Group discussion on experiences from practice (i.e., case discussion) is an ubiquitous educational activity in all types of health science education. Though common, it is also an educational activity that requires a lot from both students and teachers. Group discussions are *social* activities. The value of the activity for students partly depends on *individual* students’ engagement in the activity (Bernabeo et al., 2013; Hendry et al., 2003; Nieboer & Huiskes, 2020; Sandars, 2009; van Braak, Giroldi, et al., 2021; van Braak, Huiskes, et al., 2021). Teachers have an important task in ensuring participation to ensure educational value for *all* participants (cf. Mann, et al., 2009; van Braak et al. 2021). This task, however, can be challenging due to, for example, the open format of discussion, the large number of participants, or participant diversity (cf. van Braak et al. 2021). Each moment in the discussion potentially makes relevant for teachers a choice between alternative facilitation strategies: How much guidance do you provide? How do you monitor or adjust the process, without pre-empting valuable contributions by students in the group? For teachers, two considerations are at play here: *whether* the teacher needs to do something at a certain point in the interaction and, if so, *what kind* of intervention is called for.

Research on teacher interventions in one specific educational setting, Problem-Based Learning, provides quite a few descriptions of and suggestions for teacher interventions that are supposed to facilitate a valuable discussion—and, eventually, learning. Lee and Hin, for example, describe 48 skills of experienced tutors in facilitating tutorial dynamics. Those skills vary from “propos[ing] a method on record keeping” to “create[ing] an atmosphere so that quiet students can have a chance to speak, or provide support and encouragement when they do speak” to “stop[ping] the group from rushing to conclude the discussion of a main learning objective” (p. e938). Related work advices teachers to “think empower, not control” (Azer, 2005, p. 678), create environments that invite students to intervene (Kindler et al., 2009), take verbal and non-verbal expressions from students as “useful indices of learning” (Gukas et al., 2010, p. e10), and intervene less as the interactional competence of participants grows (Haith-Cooper, 2000; Lekalakala-Mokgele, 2010).

Although this work provides some helpful suggestions in terms of a general attitude towards students and the group discussion process, this advice is not very specific in terms of actual interventions that build such facilitative environments. For example, how exactly can one create an atmosphere in which everyone is welcome to participate, how exactly does one adapt one’s interventions to the growing interactional competence of students? We do find some literature that suggest actual teacher actions that may be helpful in this respect, for example: first leave room for participants to answer a question (to stimulate active participation; Aarnio et al., 2013), withhold evaluation of student responses (it takes back the interactional initiative, hindering collaborative elaboration of knowledge; Hmelo-Silver, 2002), ask open-ended questions (Azer, 2005), give specific process-related feedback (Kindler et al., 2009), and push for explanations, revoicing students’ turns, summarizing, and generating hypotheses (Hmelo-Silver & Barrows, 2006).

Eventually, any teacher in any specific group discussion situation needs to translate these suggestions in *context-specific and context-responsive actions*. That is, teachers need to translate generic advice into an intervention tailored to the specifics of their very interactional situation. From an interactional perspective, teacher interventions can never be done in isolation: any teacher action is done in the context of prior actions in that local interactional situation, and followed by other actions for which that teacher intervention set the scene (Schegloff, 1996; Sidnell, 2013). Teacher interventions are always occasioned by the *particulars* of the ongoing discussion. That means that the same intervention in a different interactional context can do very different things and have various effects. It also means that teacher interventions are not the sole responsibility of the teacher: they are actions that originate in *other’s* actions and contribute to the *collaborative* achievement of constructing the educational setting at hand. Finally, the fact that interventions are occasioned by prior turns means that generalized advice on the type of interventions that teachers could or ought to do falls short, as it does not recognize the local contingencies of teaching situations. So far in the literature, we find no such descriptions of teacher interventions *as they are done in their specific interactional context.* Yet, a description of the local contingencies of teacher interventions would yield a much-needed understanding of their workings *as the group discussion unfolds.* Such description would help us to understand contextual features that make an intervention relevant, to understand the entanglement of teacher and student actions, and to understand in what respect an intervention may or may not be effective given the local interactional situation.

In this study, therefore, we take a radical interactional approach to teacher interventions in group discussion. Rather than looking for generalizable heuristics about ‘good’ intervention, we systematically analyze teacher interventions in their educational ecology. In other words: Why *that* (this intervention) *now* (at this point in the discussion) (Schegloff & Sacks, 1973)? That is, we focus on the actual, observable behavior and the function of that behavior in the ongoing interaction: we describe the *actions* that teachers do when intervening, *how* these actions are done, the *timing* of those actions, and their interactional *consequences* in the ongoing interaction. The resulting description of intervening actions in their contexts provides teachers with a variety of concrete options (not action plans for guaranteed effect) to try to apply in service of tailored facilitation of unique educational group discussion situations.

## Materials and methods

### Setting

We focused our analysis on group discussions about experiences from practice (which is commonly part of ‘reflection education’; Uygur et al., 2019) in postgraduate education of Dutch General Practitioners in training. During weekly sessions lasting 60–90 min, GP residents reflect on experiences encountered in their residency (van Braak, Giroldi, et al., 2021; van Braak, Huiskes, et al., 2021; Veen & de la Croix, 2016). An experienced GP and/or a behavioral scientist/psychologist facilitates the session. Sessions usually progress through four phases (inductively identified in Veen & de la Croix, 2017):

1. one resident presents an experience in the form of telling about the situation: *telling*;
2. residents and teachers collaboratively explore the experience to clarify the situation and define a focus for discussion: *exploration*;
3. residents and teachers discuss the experience focusing on the issue defined in (2): *discussion*;
4. participants formulate the uptake (lessons learned or advice): *conclusion*.

In the following, we refer to each full cycle of (1)–(4) as a cycle of experience discussion.

### Participants

Participants were recruited by the first author via educational coordinators at the GP training institutes. The first author would email selected teachers to invite their participation (the selection ensured a representative sample over the training years). If teachers were willing to be recorded, the first author would present the research idea to their reflection group, formally asking the residents and teachers for informed consent to record their next reflection session. Only if all residents and teachers in that group gave informed consent (blinded to others in the group), the recording was pursued. On average, nine residents were present per recorded session (five minimum, fifteen maximum). Participants were in their first year (14 groups), second year (12 groups) or third year (15 groups) of training. Most group discussions (25 of 41) were facilitated by two teachers. Teachers were GPs (35), specialist physician (1) or behavioral scientists/psychologists (30) with 0.5–18 years of teaching experience. All participants consented to the recording prior to the session. Approval for this study was obtained from the Ethical Review Board of the Dutch Association of Medical Education (NVMO), dossier 829.

### Data

The data derives from 41 sessions, recorded with two or three video cameras in order to capture all participants on at least one camera. Researchers were not present during the recording. Participants were offered the option to stop the recording temporarily, but no one used this in any session. Also, up to one week after the recording, participants could ask the first author to delete parts of their recording without having to provide a reason. This option was used twice; both requests were from residents who on second thoughts did not want to have the experience they shared and subsequently discussed recorded.

To capture the verbal and non-verbal details of the interaction, the video recordings were transcribed prior to analysis using the Jeffersonian transcription conventions (see Appendix A; Hepburn & Bolden, 2013). Examples of interactional details are up- and downward arrows for up- and downward intonation changes, beak signs signaling tempo changes in speech, and underscore for stress. Examples of non-verbal details added to the transcripts are notable movements of the body and gaze direction. Recognizable personal and institutional information was anonymized in the transcripts; participant names were pseudonymized. Patient information referred to in the case discussions was never retraceable to individuals, so there was no need to anonymize this information. The resulting transcripts form the basis for analysis. Videos were used in combination with the transcripts to form a detailed understanding of what happened during the interactions.

### Analytic procedure

We analyzed the function and timing of teachers’ interventions using the data-driven, iterative process of conversation analysis (CA) (Sidnell, 2013). As an interactional, qualitative methodology, CA allows us to investigate the systematic organization of talk during the group discussions (Mazeland, 2006; Schegloff, 1968): what do participants do in interaction with each other, and how are their actions interrelated? CA is particularly suited for analyzing group discussions in educational setting because here, talk is the very gist of education (Stokoe, 2000; Watson, 1992).

The analysis was done in three phases: selection of teacher interventions from the recordings, detailed interactional analysis of those interventions, and synthesis of the analytic findings. In the first stage of analysis, the first author collected teacher interventions from all phases of the cycle of experience discussion in the 41 recordings. After consultation with the other authors, interventions were selected for analysis only if they were *non-minimal* self- or other-initiated turns at talk by the teacher (examples of minimal turns, which were excluded in this analysis, are pass-on turns like “hm mm” and listener tokens like “yes” and “ok”). Discussing instances of this collection in data sessions (Sidnell, 2013) with the author group and external CA researchers, we chose to focus on the *first* non-minimal teacher turn at talk in each cycle of experience discussion. More than subsequent interventions, teachers’ *first* turns display an urgency for action. Note, though, that those first turns do not necessarily occur early in the cycle of experience discussion. They may also occur later in the cycle, sometimes even in phase (3), the discussion.

In the second stage of analysis, the authors collaborated to conduct a detailed interactional analysis of all first teacher turns in the 142 cycles of experience discussion in our data by describing for each intervention:

- the *actions* done with it (e.g., to correct, to explain, to corroborate, to invite for elaboration);
- how these actions are occasioned at that point in the interaction (i.e., how they come about at this point in the ongoing interaction, so their *timing* in relation to the directly preceding interaction and to the four phases)
- *how* these actions are done (e.g., corroborating a conclusion, which is the action, by illustrating it with an own experience, which is *how* it is done);
- and the *effect* of those actions on subsequent interaction (i.e., what happens in the next turn, directly following the teacher intervention?).

Disagreement on analytic details was solved in close consultation within the author group; analytic findings at this stage were discussed with external CA researchers in data sessions (Sidnell, 2013) to validate our initial analysis and gain complementary analytic insights. The level of detail in this analytic phase helped us to understand “the resources, practices, procedures and reasoning on which the participants themselves rely in accomplishing particular actions in and in making sense of the contributions of others” (Heath et al., 2007).

In the third stage of analysis, the authors synthesized the detailed interactional findings by inductively categorizing the actions with similar functions into broader *types* or *categories* of actions. We discussed the synthesized findings with external researchers in presentations. For an extensive account of the rigor of such analytic procedure, see Sidnell (2013, p. 86–98).

## Results

From our detailed interactional analysis, it became evident that the timing of first teacher interventions is non-random. Also, the type of actions done with the interventions is related to their timing: certain actions are typically done in a particular phase of the cycle of experience discussion. In the following, we will first present a few overarching observations about the timing and types of actions done by teachers’ first turns. Next, we show in detail how these are done and reacted to in the different phases of the experience discussion cycle.

### Overarching observations on the *timing* of teachers’ first turns

In principle, teachers can take a turn at talk at any point in the cycle of experience discussion. Yet, our analysis revealed that teachers tend to first take a turn between telling (1) and exploration (2), and in the exploration (2) and –mainly—discussion (3) phases—see Table 1 below. Interventions were less frequently done in the telling phase, and no interventions were done in the conclusion (4) phase. Within each phase, teachers’ first turns tend to be positioned at ‘points of no return’: the last possible opportunity to contribute to an otherwise completed unit of interaction (Schegloff & Sacks, 1973).

**Table 1:** Examples and timing of teachers’ moderating, expert, and evaluating actions

Moderating Actions (n = 95) When teachers moderate the interactional process to refocus it topically or in terms of participation		
invite elaboration for complete telling (n = 45)	“But- bec- because you then feel like ‘hey I’m running out of time’ or ‘it’s spilling over’ and is that then- what erm- how- how you experience it then?” (B815; see Excerpt 1)	n = 19 in telling phase n = 26 in exploration phase
focus discussion (n = 18)	“Would you mind zooming in on that?” (C811; see Excerpt 2) “We:- wait a bit. ((looks up)) I’d like to jus- because it’s more like the cherry on top, I’d like to uh wait a bit with that one.” (E812; see Excerpt 3)	n = 3 in telling phase n = 14 in exploration phase n = 1 in discussion phase
request clarification for clear telling (n = 12)	“What do you mean by that?” (E915)	n = 10 in telling phase n = 2 in exploration phase
create room for non-teller contributions (n = 6)	“And what should be the goal of that?” (H825) “I erm- [name resident], what do you want to say, what’s your view on this?”(B859)	n = 6 in discussion phase
initiate meta-perspective on discussion (n = 4)	“But what was happening here?” (A715)	n = 4 in discussion phase
direct interaction to group (n = 4)	“You can tell the group.” (H825) “Do you recognize that?” (directed at group) (C808)	n = 1 in telling phase n = 3 in discussion phase
direct interaction back to one speaker (n = 2)	“Shall we keep the focus central for a while, guys?” (B869)	n = 1 in telling phase n = 1 in discussion phase
nominate topic (n = 2)	“And uh (.) what struck me is that at that moment you were in doubt and that the husband actually (.) uh cut the- through the knot, like now I must resuscitate.” (G824; see Excerpt 4)	n = 2 in discussion phase
formulate uptake/gist (n = 2)	“So, here, giving information is key” (D887)	n = 1 in exploration phase n = 1 in discussion phase

Expert actions (n = 22) When teachers provide input while constructing their position as having advanced access to the ways of being and doing in the GP profession		
suggests possible future action/solution (n = 8)	“Do you know the KNMG’s standard note? On giving medical certificates?” (D887)	n = 2 in telling phase n = 1 in exploration phase n = 5 in discussion phase
provide interpretation of medical situation (n = 6)	“Could the mourning process still be at play here?” (B859)	n = 1 in telling phase n = 2 in exploration phase n = 3 in discussion phase
challenge a way of doing (n = 2)	“That about the benzo’s- I don’t know for sure, I think that is more an issue of (…)” (B851)	n = 2 in discussion phase
offer a heuristic (n = 2)	“And if you’re [doing] the initial screening, at [point] A and B, if someone at that point- like ‘hey, there’s no breathing, and I can’t feel a pulse’ well, then there’s no need for doubt. Then you just jump onto them.” (G824; see Excerpt 4)	n = 2 in discussion phase
align with a presented way of doing (n = 2)	“I actually also agree with your idea that it doesn’t matter much. I think that oftentimes it is a complex presentation in practice.” (A851)	n = 1 in telling phase n = 1 in discussion phase
reassure (n = 1)	“But I think- I just wanted to say- do you think it’s weird? That happens to me too, even after twenty years.” (A823)	n = 1 in telling phase
explain (n = 1)	“Uhm (.) you’ve got uhm (.) four compartments in your calf (…).” (A851)	n = 1 in discussion phase

Evaluating actions (n = 10) When teachers normatively judge the telling or the reported conduct		
ratify professional conduct (n = 5)	“yes, listening to your story, it sounds utterly adequate for this [case]. It was good that you instantly saw like well, I’ll follow ABCD (…).” (G824; see Excerpt 4)	n = 1 in telling phase n = 3 in exploration phase n = 1 in discussion phase
appraise telling as material for discussion (n = 4)	“I think that it’s a very good topic, worth discussing for a while here.” (A852)	n = 4 in exploration phase
compliment teller for way of telling (n = 1)	“I think also that you tell it very nicely.” (A851)	n = 1 in exploration phase

N.B. Since first turns sometimes featured more than one action and some actions were ambiguous in terms of the three glosses, the total number of instances does not equal the total number of analyzed first teacher contributions

### Overarching observations on the *actions* done with the teachers’ first turns

We first identified the actions done by teachers’ first turns in each cycle of experience discussion. Some actions intervened in the interactional process itself, such as *focusing the discussion, requesting clarification,* and *nominating a topic*. These we glossed as moderating actions, since they mediate in the interactional process to refocus it topically or in terms of participation. Moderator actions are most frequent during the experience telling (phase 1) and the exploration (phase 2)—see Table 1.

Some actions contribute to the collaborative achievement of educational value by providing input from an epistemically higher position, such as *suggesting possible future actions, offering a heuristic,* and *reassuring*. We categorized these as expert actions, since the teacher with those actions can be seen as constructing their position as having advanced access to the ways of being and doing in the GP profession. Expert actions typically occur in the discussion phase (3).

Finally, we found a variety of *assessments*. These we categorized as evaluating actions, because they normatively judged the telling or the reported conduct. Such actions are done at rather specific moments, usually directly after the telling (1).

Comparing these three overarching categories, we observed that moderating actions are least ‘steering’, in the sense that they may *direct* the interaction but do not explicitly and normatively *address* the topic at hand. Expert actions have a normative dimension in the sense that they orient to the hierarchical relationship between ‘beginner’ and ‘advanced’ ways of dealing with a situation. Evaluating actions are most directive given their normative nature and the way they limit the options for subsequent actions.

An overview of the types of actions, specific examples, and their occurrence per phase is presented in Table 1.

Following Table 1, we provide a detailed analysis of four teacher interventions mentioned in Table 1. These four examples are from different reflection sessions. We present them in order of the phases of the experience discussion cycle (except the conclusion phase, where teachers’ first turns did not occur). Our aim here is not to be exhaustive, but to illustrate how the different actions in their interactional context construct the teacher as moderator, expert and evaluator in this educational group discussion setting. An overarching analytic observation throughout these examples is that teachers’ first turns, no matter the phase they are done in, treat the preceding interaction as in need of correction or direction to add educational value.

### First turn during the telling: do we have a complete and clear experience telling?

Teachers’ first turns in the telling phase are predominantly *moderating* actions. They tend to orient on a lack of *completeness* and *clarity* of the information shared so far. This orientation is displayed in moderating actions like *inviting elaboration for complete telling* and *requesting elaboration for clear telling* (see Table 1). Both actions influence what is ‘at the table’ for later discussion.

Excerpt ﻿1 illustrates a moderating action of the teacher (T). With this action, he treats resident R1’s telling as incomplete. The resident has just started her first internship at a busy GP practice. She notices that patients are scheduled for consultation with her outside usual consultation hours. The patients are scheduled in her agenda without her agreement. That frustrates her, and she shares her frustration with the group. So far, the telling has been a factual description of the situation, summarized by the resident in lines 1–12. Her summary projects a next item in a list (“that is one”) (Selting, 2007), but in overlap with that, the teacher redirects the telling in terms of its topic: he both pre-empts the residents’ continued telling and invites further telling on another issue (feelings associated with the situation).


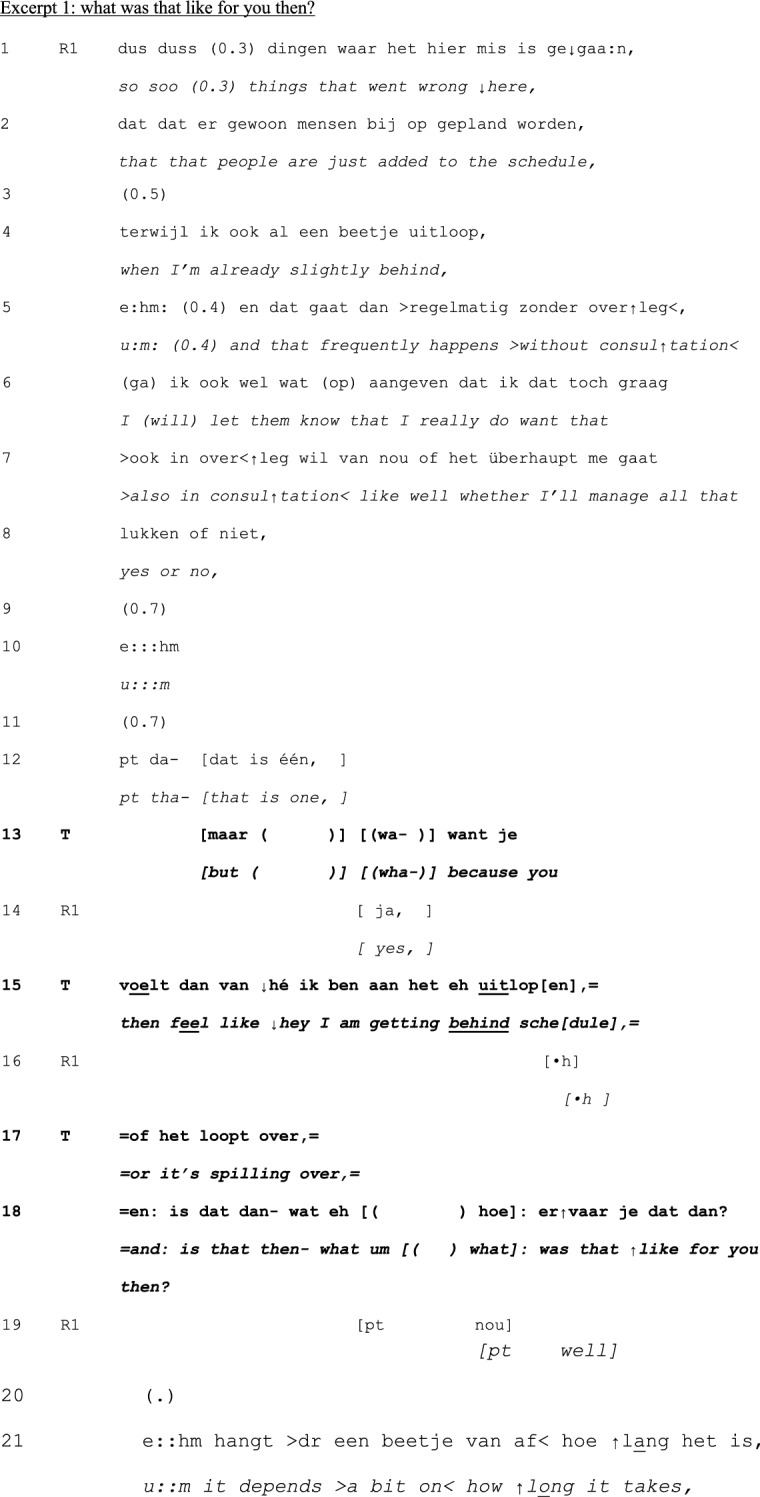


For participants other than the teller resident, the projected closing of the first part of this telling (line 12) provides a last opportunity to say anything related to what R1 has shared so far. In the transition space between the teller’s conclusive statement and a possible next turn (lines 11–13; Sacks et al., 1974), T takes a turn and invites the teller to elaborate on her emotions. Stress on “feels” (line 15) and “experience” (line 18) underscores the contrast between already shared facts and thus far unshared feelings. While the format of T’s request for elaboration on feelings connects to the prior turn (see, for example, the loosely quoted “I’m getting behind” in line 15 and the connector “and” in line 18), it simultaneously makes talk about *other* matters relevant (Jefferson, 1984; Walker, 2007). This *topical shift* displays moderating work.

Sometimes, moderating actions not only *shift the topic* of the telling, but *pre-empt further telling* by pivoting to the subsequent exploration phase. Such pivots bridge the telling and exploration by connecting to the telling while having independent topical potential to create room for others’ contributions (Holt & Drew, 2005). Excerpt 2 shows an example of this pivoting function of moderating actions: teacher T transforms a ‘pit stop’ in the telling into an opening for further exploration of the experience by the group. In Excerpt 2, we join the group at a possible end to the telling of resident R1. She has shared an experience from a shift as GP in a nursing home. Towards the end of that shift, around dinner time, she wanted to see two patients. These patients, however, were already in the dining room, so the nurses resisted her request to have the patients come in for consultation at that moment. Resident R1 solved the situation by taking one of the two patients for consultation. She presents this situation as a first example of a bigger issue (line 1), the issue being her (lack of) ability to stand up for her own agenda despite the risk of being unfriendly to others (lines 3–5). Again, this “first example” projects a second example (Jefferson, 1990; Selting, 2007)—and thus continuation of the telling. At the same time, R1’s summary assessment of the first example is recognizably closure-implicative (i.e., it can be recognized as rounding off the telling so far; Hoey, 2018). T in response to that (Arminen, 2005) then transforms the telling into an opportunity for exploration of the shared situation (lines 12–29):


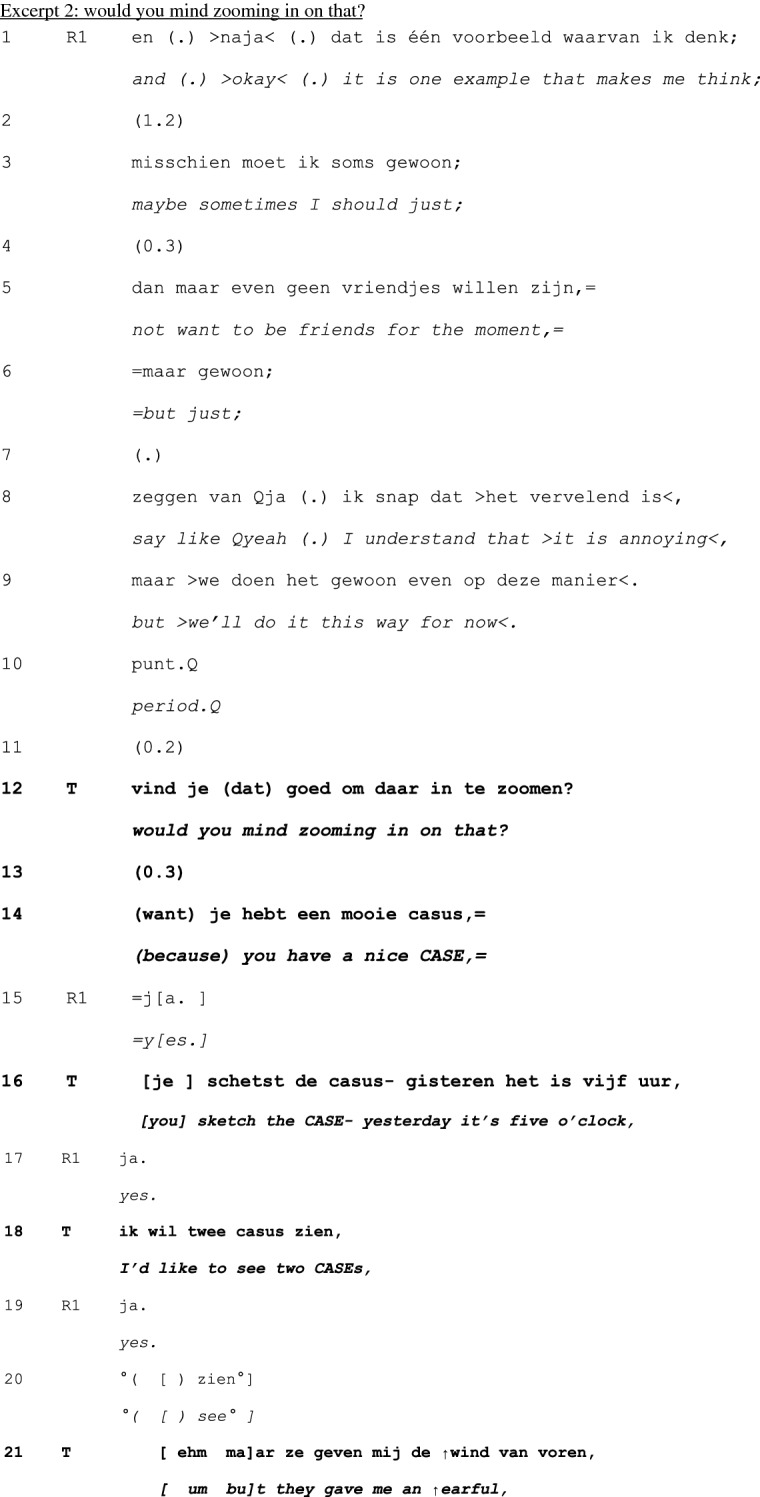

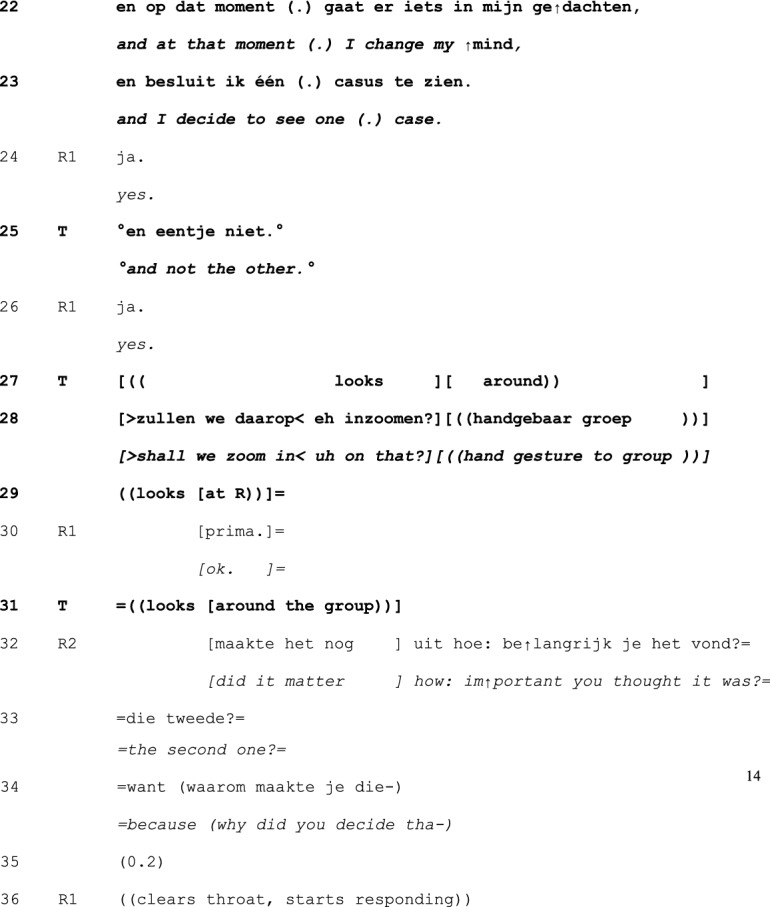


As in Excerpt 1, the timing of the teacher’s self-selection in line 12 is salient. It is done when R1 pauses but signals that there is more to come in the presentation (lines 1–10). At that point, T produces a summary of key points from the telling so far (lines 16–25). This summarizing formulation (Solem & Skovholt, 2019) functions as a proposal for a focus for discussion for ratification by the teller (line 29) and group. With that proposal, he treats the telling so far as complete, clear and focused enough to move on to the exploration phase. R1 accepts the proposal, with which she opens the floor for exploration. T then looks around, indeed inviting the group to react to the proposal (see Bjork-Willen & Cekaite, 2017 and Willemsen et al., 2018 on the “lighthouse gaze” used by the teacher). By starting to ask a question (lines 32–34), one resident accepts the invitation and starts the exploration phase.

So, in Excerpt 2, the teacher (1) treats the telling as sufficient (complete, clear) for discussion even if R1 could have continued, (2) directs the interaction topically by highlighting particular elements for discussion, and (3) changes the participation frame from individual to group. In collaboration with the teller’s and groups’ validation and acceptance, these actions moderate the interaction by progressing it forward in terms of phase, topic, and participation frame.

### First turn during exploration: do we have all and only all relevant information for a focused discussion?

Teachers’ first turns in the exploration phase orient to the *importance of establishing a focus for discussion* and to the *relevance of resident questions to the issue at hand*. This orientation is mainly visible in actions which *invite elaboration for a complete telling* and *focus the discussion*. These are *moderating* actions that make the specific situation shared by the teller reactable for everyone, with a specific focus for discussion to avoid too much meandering. A few first turns during exploration phases are *evaluating* actions, with which the teacher judges medical conduct or the telling of that conduct. Examples of that are when teachers *ratify professional conduct* shared in the telling, *appraise the telling as material for discussion*, or compliments the teller with their way of telling (for examples, see Table 1).

In Excerpt 3, we show a *moderating* action at the start of the exploration phase. The resident presenting his experience (R1) in this Excerpt formulates a topic for exploration in the group (line 1–2). He has just told that he had recently done a physical examination on a patient’s neck, where the patient had noticed some irregular feeling. While doing the examination, the resident became strongly suspicious of cancer. He was in doubt whether to tell the patient during the consultation, or wait for the scan results which he ordered. In the end, he only very implicitly shared his suspicions with the patient. In Excerpt 3, he shares his doubts about that strategy: “so that bothers me a bit like (.) how (.) could I have brought this across the best” (line 1–2). Following this concluding statement and the teachers’ display of “preparedness to move onto some next-positioned matter” (with the “okay”; see Hoey, 2018, p. 332; Beach, 1993), fellow resident R2 initiates the exploration phase by posing an exploratory question (line 7). The unfolding of interaction, however, is pre-empted by a moderating action of the teacher (lines 8–11):


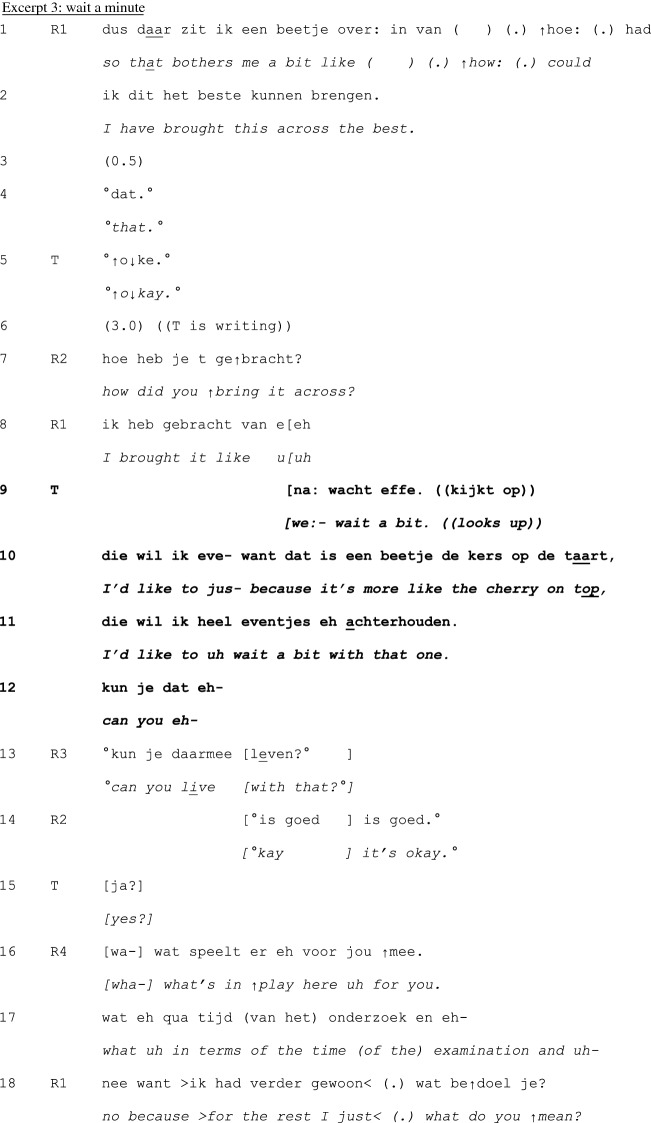


The first exploratory question invited the teller (R1) to share their solution to the issue (line 7). T, however, self-selects in the middle of R1’s turn (line 9; Clayman, 2013), halting the narrative before the report reaches its conclusion. The timing of this action displays a sense of urgency, which the teacher accounts for by treating the posed question as touching upon the crux of the shared experience, something that is best left for later in the discussion. The teacher’s action is pre-emptive, but unlike the actions in Excerpt 1 and 2, does not direct the interaction towards a specific topic. In that sense, the teacher here indeed redirects the exploration without determining its course. With R4’s subsequent question (line 16), which accepts the topical redirection, the exploration phase is reopened on a different track. The moderating work is now complete.

### First turn during discussion: is the discussion so far focused towards and sufficient to answer the issue?

Teachers’ first turns in the discussion phase orient to the *importance of a diversity of perspectives and solutions that address the issue under discussion*. This orientation is visible in *moderating* actions that direct the interaction back to the focus and invite others to share their views. Examples of such actions are *creating room for non-teller contributions* and *directing the interaction back to the group.* Teachers’ orientation to the *importance of a diversity of perspectives and solutions that address the issue under discussion* is also visible in *expert* actions that provide input from a position of advanced access to professional norms relevant to the issue being discussed. Examples of such actions are *suggesting a possible solution, providing an interpretation of the situation discussed,* and *offering a heuristic.* Though less normative than evaluating actions (of which we only see one in this phase), expert actions do have normative (i.e., judgmental) flavor to them.

Many first turns in the discussion phase are multifunctional: they do more than one action, and those actions are of different types. In Excerpt 4, we show a detailed example of a relatively late first teacher turn that includes actions that construct *all three* teacher roles: moderator, expert and evaluator. The late timing of this intervention shows a teacher orientation on the primacy of the group in participating in the interaction.

Preceding the interaction presented in this Excerpt, R1 has shared an experience from an out-of-hours shift. The shift had just started when a female patient came in, who just shortly after coming in appeared to lose her heartbeat. The resident started CPR, together with the patient’s partner. Only shortly afterwards, however, the woman ‘came back’ and appeared to have been hyperventilating. In the preceding interaction, R1 and the group have critically discussed R1’s decision to start CPR. At a transition relevant place in that interaction, the teacher T takes a turn (line 10; the teacher has not said anything during telling and exploration). Here, he offers a compliment, a heuristic, and opens up a new subtopic—thus doing moderating, expert and evaluator work all at the same time.


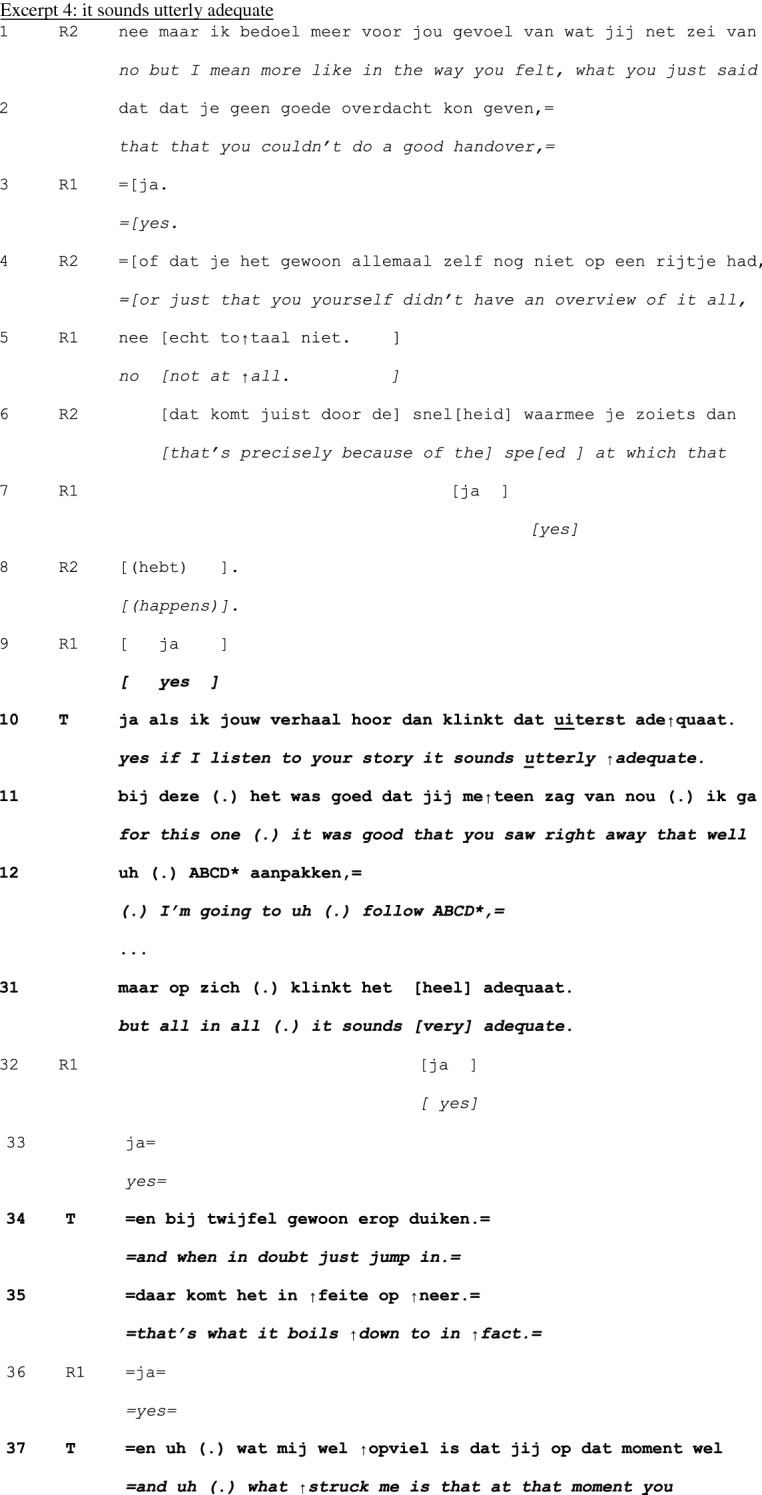
 
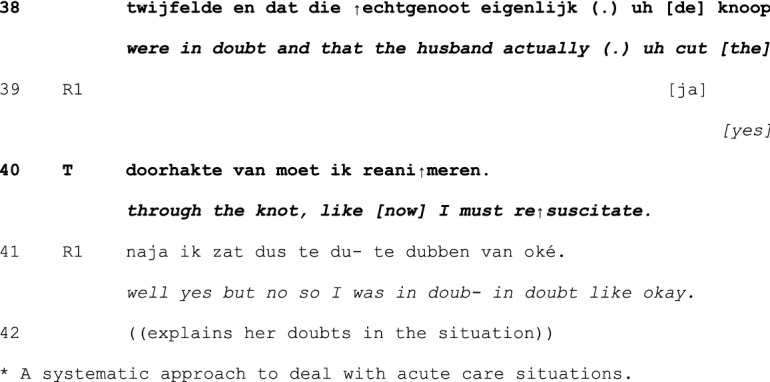


R1 and R2 are discussing a factor that may have contributed to R1’s feeling of inadequacy and incompetency in this situation (lines 1–8). When T takes a turn (line 10), he produces an extremely positive assessment of R1’s conduct, an *evaluating* action. The positive assessment contrasts with the discourse of *inadequacy* preceding it and nips any search for potential causes of the doubt in the bud. The combination of reaffirming (lines 11–20) and repeating (line 31) the assessment, as well as presenting an expert professional norm (lines 34–35; an *expert* action) settles the issue of doubt about R1’s conduct. At least, it is not contested after the teacher’s contribution and thus collaboratively established as concluded.

With these evaluating and expert actions, the teacher reframes the *prior* discussion. T’s next action (formatted as an additional comment, lines 37–40) functions as a pivot (Holt & Drew, 2005) to *further* discussion on an as yet undiscussed aspect of the experience. In that sense, it does *moderating* work in terms of the topic of following interaction. Coming back to prior issues after such topic proposal would likely require interactional work (Sirois & Dorval, 1988), which means that this moderating action limits the type of actions that can be done by others in subsequent interaction. In conclusion, this relatively late teacher intervention, which took only few seconds, still has the potential to leave a mark on large parts of preceding *and* subsequent interaction.

## Discussion

In this study, we described the timing and functions of actions teachers do when intervening in group discussion in postgraduate medical education. A first main finding is that teachers’ do moderating, expert and evaluating types of actions. Second, these actions show evidence of responding to something in need of action in the ongoing interaction: the telling is not complete, clear or focused, the exploration is not relevant, the discussion does not include different perspectives or does not help find ways to deal with the issue. Interventions are embedded in and occasioned by ongoing interaction. With indexical placement and explicit references to prior turns, teachers produce the interventions as occasioned by prior turns. Residents’ uptake in the form of ratifying the moderating, expert and evaluating actions done with the interventions constructs the interventions as a collaborative interactional accomplishment.

The observation that teachers in their turns orient to something in need of action has implications for ‘doing teaching’ in this context. First, if teachers *do* intervene, the interactional meaning of their actions that is constructed with those interventions is that the teacher deems intervention necessary. Second, if teachers *do not* intervene, the constructed meaning is an implicit ratification of the activity at hand (Nieboer et al., 2020)—although the absence of a teacher action could also be treating the context as one where residents and teachers have particular interactional rights. In combination, these interpretations imply that each teacher intervention displays an *interpretation of the preceding interaction*. What the teacher does could be seen as more than ‘just listening’ like any other participant (described as the participant role of teachers; Veen & de la Croix, 2016): whatever the teacher does or does not do constitutes a normative response to and in the ongoing interaction.

The types of actions described here could be interpreted as enactments of three different teacher roles: that of a moderator, an expert, and an evaluator. In health science education literature, the medical teacher has been described as acting as an information provider, role model, facilitator, assessor, planner, and resource developer (Harden & Crosby, 2000). These roles partially overlap with those identified in our study: the facilitator role resembles actions done by the teachers in a moderator role, information provider, role model and resource developer resemble actions teachers do as experts, and the assessor role resembles the actions done as evaluator. The current study’s specific description of these roles in actions that teachers do when enacting those, thus adds a new dimension to the knowledge about roles of medical teachers. Also, the detailed description of authentic interactional situations corroborates and portrays in detail how the ambiguity of role enactment, for example as experienced by physician-mentors in clinical practice (Meeuwissen et al., 2019), plays out in practice (see especially Excerpt 4). That gives us suggestions for future practice, which we will outline shortly.

A second main finding relates to the timing of teacher interventions. First teacher interventions are done at critical moments in all phases but the concluding one. Only rarely are they done when a resident presents their experience and even then mostly towards the end of the phase. Expert and evaluating actions, which are relatively limiting compared to moderating actions in terms of the type of actions that can be done in subsequent interaction, have a close temporal link to the later phases of the reflection discussion cycle. We could interpret these findings as hinting at an orientation of teachers to intervene as little and late as possible. Such interpretation would be in line with a guideline reported in early literature on teacher roles in group discussion (Gall & Gall, 1993, p. 11): “do not intervene in the groups any more than is absolutely necessary”. More generally, the proposed connection between the timing and type of teacher interventions is a specification of the general role descriptions of medical teachers mentioned earlier (Harden & Crosby, 2000; Meeuwissen et al., 2019; Stoddard & Borges, 2016).

### Practical implications

Based on our analysis, we offer three practical suggestions for teachers. First, teachers could use the pivotal function of interventions strategically. If the interaction is valuable in terms of established educational aims, teachers do not need to intervene—even if the discussion takes an unexpected turn. Later interventions can equally well leave a mark on the interaction so far while leaving plenty of room for the participants to explore and discuss (see Excerpt 4). Research in another educational setting has shown that bridges between a current and new topic progress the interaction, and thus move the discussion forward (Creider, 2020). Second, teachers could approach the question of intervention from a role or identity perspective. In situations where teachers do feel the need to intervene, they could briefly consider how a moderating, expert or evaluating action would contribute to the interaction: is pivoting the ongoing interaction necessary? Would that require mere moderation, expert input, or evaluator feedback? Would that require instant involvement, or could it also be done later? Such considerations take time and may draw attention away from ongoing interaction. Explicitly marking one’s considerations by sharing them out loud may be an initial strategy to create that time and attention. Teachers could also practice with video-recordings of actual group discussion to create opportunities to literally halt the interaction and discuss their considerations with a co-teacher. Third, teachers could consider how ‘multi-role’-interventions could be a way to deal with the ambiguity of role enactment. In a given situation, enacting more than one role could contribute to the session aims more than avoiding the one or the other to avoid ambiguity. Even when not clearly marked, interaction can move smoothly when teachers’ interventions are multifunctional—as we have seen in Excerpt 4.

### Strengths and limitations

Reformulating the considerations of intervention in interactional terms refocuses our attention on behaviors that constitute the course of actual interaction. Using conversation analysis to focus on the ‘why that now’ (Schegloff & Sacks, 1973) of actual teacher behaviors in ongoing interaction, enabled us to provide detailed descriptions of the interventions in terms of timing and actions enacted by teachers at various phases in interaction. Theoretically, the synthesis of actions in moderating, expert, and evaluating teacher roles is a valuable addition to our current understanding of facilitation of group discussion. Methodologically, the detailed conversation analyses of actual teacher behavior in specific, authentic educational moments, show how conversation analysis can benefit understanding of education as an interactional enterprise: certain social actions build certain educational roles, and certain educational roles can be enacted for participants by doing certain social actions. In that way, participants collaboratively build educational value *as the interaction unfolds*. Practically, the detailed analyses can form the basis for teacher training, suggesting a diverse palette of possible actions to construct various teacher roles. Showing teachers exemplary situations for collaborative discussion of what happens in these situations can help teachers understand how possible action trajectories could lead to different interactional outcomes (Stokoe & Sikveland, 2017).

Our study is limited in two respects. First, we could not analyze situations where teachers decided *not* to intervene. Such considerations are not observable and are not likely to be reliably remembered after the fact. Future research could analyze situations where teachers’ embodied behavior shows evidence of possible doubts to jump in. Second, moderating, expert and evaluating actions may not be equally relevant in educational contexts where socialization into a *profession* is not the main aim. The principle behind these action categories, however, would likely be similar: teachers in other contexts may also do particular actions that construct their various roles or identities at specific moments in that interaction and make their interventions analyzable in terms of the timing, form, and effect of these actions.

Overall, this conversation analytic study provides a unique level of detail to descriptions of what teachers do and participants collaboratively construct their various roles in group discussion settings. Highly detailed interactional analyses like those reported in this article are supplementary to for example observational approaches like video reflexive ethnography, which are limited with regards to describing exactly how social actions interrelate and construct the interactional context *as the interaction unfolds.* Such approaches, however, could be beneficial for gleaning teacher interpretations of their own behavior (see for an example in the context of group discussion: van Braak et al. (2022).

Though detailed and related to specific instances of interaction, the analyses offer generalizable insights into a variety of useful intervention strategies in group discussion setting. The researched setting is quite unstructured, with few format restrictions and little teacher guidance. When the group discussion format is stricter, the need for moderating actions may probably be low (or the other way around, high, if students defer from the prescribed format). Depending on the educational principles and institutionally assigned tasks for teachers in differently structured group discussions, teachers may enact expert and evaluating actions in different ways or at different moments of the interaction. Still, comparing our current findings to earlier, more general, investigations of teacher facilitation in group discussion setting, we have no reason to believe that moderating, expert and evaluating actions are specific to this particular group discussion format in this particular GP setting. As such, this research contributes to addressing the challenge of facilitating group discussions in the context of health science education and beyond.

## Appendix A

Transcription conventions (based on Hepburn & Bolden, 2013).

**Table:**

Symbol	Meaning
[word [word	Overlapping talk
word = = word	‘latching’: no gap between two turns
(1.0)	Pause of 1.0 s
(.)	Micro pause, shorter than 0.2 s
?	Turn is produced with sharp rising intonation
,	Turn is produced with slightly rising intonation
—	Turn is produced with flat intonation
.	Turn is produced with falling intonation
↑ ↓	Marked rising or falling shift in syllable intonation
WORD	Louder than surrounding talk
‘word˚	Softer than surrounding talk
word	Stressed syllable
wo:rd	Lengthening of the preceding sound
wo-	Abrupt cut-off
> phrase <	Faster than surrounding talk
< phrase >	Slower than surrounding talk
hh	Audible aspiration
.hh	Audible inhalation
(word)	Unclear talk
( )	Inaudible talk
((points))	Verbal description of (non-verbal) actions
